# Nonadjuvanted Bivalent Respiratory Syncytial Virus Vaccination and Perinatal Outcomes

**DOI:** 10.1001/jamanetworkopen.2024.19268

**Published:** 2024-07-08

**Authors:** Moeun Son, Laura E. Riley, Anna P. Staniczenko, Julia Cron, Steven Yen, Charlene Thomas, Evan Sholle, Lauren M. Osborne, Heather S. Lipkind

**Affiliations:** 1 Division of Maternal-Fetal Medicine, Department of Obstetrics and Gynecology, Weill Cornell Medical College, New York, New York; 2 Department of Obstetrics and Gynecology, Weill Cornell Medical College, New York, New York; 3 Department of Information Technologies & Services, Weill Cornell Medical College, New York, New York; 4 Division of Biostatistics, Department of Population Health Sciences, Weill Cornell Medical College, New York, New York; 5 Division of Health Informatics, Department of Population Health Sciences, Weill Cornell Medical College, New York, New York; 6 Department of Psychiatry, Weill Cornell Medical College, New York, New York

## Abstract

**Importance:**

A nonadjuvanted bivalent respiratory syncytial virus (RSV) prefusion F (RSVpreF [Pfizer]) protein subunit vaccine was newly approved and recommended for pregnant individuals at 32 0/7 to 36 6/7 weeks’ gestation during the 2023 to 2024 RSV season; however, clinical vaccine data are lacking.

**Objective:**

To evaluate the association between prenatal RSV vaccination status and perinatal outcomes among patients who delivered during the vaccination season.

**Design, Setting, and Participants:**

This retrospective observational cohort study was conducted at 2 New York City hospitals within 1 health care system among patients who gave birth to singleton gestations at 32 weeks’ gestation or later from September 22, 2023, to January 31, 2024.

**Exposure:**

Prenatal RSV vaccination with the RSVpreF vaccine captured from the health system’s electronic health records.

**Main Outcome and Measures:**

The primary outcome is preterm birth (PTB), defined as less than 37 weeks’ gestation. Secondary outcomes included hypertensive disorders of pregnancy (HDP), stillbirth, small-for–gestational age birth weight, neonatal intensive care unit (NICU) admission, neonatal respiratory distress with NICU admission, neonatal jaundice or hyperbilirubinemia, neonatal hypoglycemia, and neonatal sepsis. Logistic regression models were used to estimate odds ratios (ORs), and multivariable logistic regression models and time-dependent covariate Cox regression models were performed.

**Results:**

Of 2973 pregnant individuals (median [IQR] age, 34.9 [32.4-37.7] years), 1026 (34.5%) received prenatal RSVpreF vaccination. Fifteen patients inappropriately received the vaccine at 37 weeks’ gestation or later and were included in the nonvaccinated group. During the study period, 60 patients who had evidence of prenatal vaccination (5.9%) experienced PTB vs 131 of those who did not (6.7%). Prenatal vaccination was not associated with an increased risk for PTB after adjusting for potential confounders (adjusted OR, 0.87; 95% CI, 0.62-1.20) and addressing immortal time bias (hazard ratio [HR], 0.93; 95% CI, 0.64-1.34). There were no significant differences in pregnancy and neonatal outcomes based on vaccination status in the logistic regression models, but an increased risk of HDP in the time-dependent model was seen (HR, 1.43; 95% CI, 1.16-1.77).

**Conclusions and Relevance:**

In this cohort study of pregnant individuals who delivered at 32 weeks’ gestation or later, the RSVpreF vaccine was not associated with an increased risk of PTB and perinatal outcomes. These data support the safety of prenatal RSVpreF vaccination, but further investigation into the risk of HDP is warranted.

## Key Points

**Question:**

Is there an association between nonadjuvanted bivalent respiratory syncytial virus prefusion F (RSVpreF) protein subunit vaccination during pregnancy and preterm birth?

**Findings:**

In this cohort study of 2973 patients who delivered during the 2023 to 2024 recommended vaccination period, 34.5% had evidence of prenatal RSVpreF vaccination. There was no increased risk of preterm birth based on maternal vaccination status.

**Meaning:**

These clinical vaccine data add to the existing evidence from trials supporting the safety of prenatal RSVpreF vaccination.

## Teaser

This cohort study examines whether there is an association between prenatal respiratory syncytial virus (RSV) vaccination with a nonadjuvanted bivalent RSV prefusion F protein vaccine, preterm birth, and other perinatal outcomes.

## Introduction

In the United States, respiratory syncytial virus (RSV) contributes to 58 000 to 80 000 annual hospitalizations and 100 to 300 annual deaths in children younger than 5 years.^1,2,3^ Two strategies were recently approved by the US Food and Drug Administration (FDA) for RSV infection prevention in infants: (1) seasonal administration of a nonadjuvanted bivalent recombinant RSV prefusion F (RSVpreF) protein subunit vaccine (Pfizer)^4^ to pregnant individuals or (2) postnatal nirsevimab (monoclonal antibody) for infants aged up to 8 months.^5,6,7^ For the 2023 to 2024 RSV season, the limited supply of nirsevimab^8^ made prenatal vaccination more important.

On September 22, 2023, the US Centers for Disease Control and Prevention (CDC)’s Advisory Committee on Immunization Practices recommended the RSVpreF vaccine to be administered to most pregnant individuals from September to January in the continental United States.^9^ This is in contrast to the RSV adjuvanted vaccine (GlaxoSmithKline), which is not approved for use in pregnant individuals based on a trial that was terminated early due to an elevated risk of premature birth and associated neonatal deaths.^10^ Although the RSVpreF vaccine was approved by the FDA,^11^ the gestational age window was limited to 32 0/7 to 36 6/7 weeks due to concerns raised about the numerical difference seen in preterm birth (PTB) among participants who received the RSVpreF vaccine from 24 to 36 weeks’ gestation. Of note, the imbalance was only seen in participants residing in low- to middle-income countries.^4^

The recommendation that most pregnant individuals, with few exclusions, should receive the RSVpreF vaccine between 32 0/7 and 36 6/7 weeks’ gestation was endorsed by major US obstetric care professional organizations.^12,13^ However, clinical data from the US 2023 to 2024 RSV season are currently lacking. Therefore, we aimed to examine the uptake of RSVpreF vaccination among a medically and demographically diverse pregnant population during the 2023 to 2024 RSV season and compare those who were prenatally vaccinated with those who were not.

## Methods

This is a retrospective observational cohort study of patients who delivered at 32 0/7 weeks’ gestation or later at 2 New York City (NYC) hospitals within 1 health care system from September 22, 2023, to January 31, 2024. We chose this gestational age threshold to include patients who had an opportunity to be exposed. Patients with unknown gestational ages and those with multifetal gestations were excluded. Data were extracted directly from a research data repository populated with structured data from the electronic health record (EHR). This repository is updated on a regular basis with a 1-week lag time and captures standardized and templated fields, including recorded diagnoses (*International Classification of Diseases, Tenth Revision, Clinical Modification* [*ICD-10-CM*]) and billing codes (*Current Procedural Terminology* and Healthcare Common Procedure Coding System) as well as data on visits, medications, and laboratory and imaging results. The repository leverages existing institutional infrastructure^14^ and is subject to rigorous quality assurance checks by informatics personnel and clinical staff. Data were extracted using direct structured query language queries against the repository by 2 of us (S.Y. and E.S.) and validated by 2 of us (M.S. and A.P.S.). The EHR links birthing parent and infant records in the obstetric module (Epic STORK). Demographic information such as race and ethnicity were extracted from the demographic fields, which are typically captured by self-report during visit encounters. Race and ethnicity were collected because the burden of RSV illness^15^ and the likelihood of vaccination^16,17^ are disproportionate among different racial and ethnic groups. Estimated delivery date (EDD) and gestational age were identified through templated fields; it is institutional practice to enter the EDD in the EHR using the best available clinical estimation.^18^ This study was approved by the Weill Cornell Medicine institutional review board with a waiver of informed consent due to it having public health benefit, posing minimal risk to patients, not being practical to carry out without a waiver, and not adversely affecting the rights and welfare of participants due to its retrospective nature. We report our findings following the Strengthening the Reporting of Observational Studies in Epidemiology (STROBE) reporting guideline.^19^

The study exposure is evidence of prenatal RSVpreF vaccination. EHR documentation of vaccinations was captured through multiple channels: (1) recorded after on-site vaccine administration at any health care system–affiliated clinical site, (2) fed into the EHR if vaccine was administered at a different health care system within Epic Care Everywhere and the information was reconciled by clinical staff, (3) fed into the EHR if the vaccine was administered at a pharmacy owned by a pharmacy benefit manager (PBM), (4) electronically prescribed to a non-PBM pharmacy and reconciled by the pharmacy, and (5) manually recorded by staff based on patient-reported vaccination externally. Patients vaccinated at 37 weeks’ gestation or later (n = 15) were classified as being unvaccinated because they were not at risk of having a PTB.

The 2 included hospitals were chosen because they were the earliest in our system to adopt on-site administration of the RSVpreF vaccine and therefore had the largest number of immunized patients to maximize study power. At New York Presbyterian Hospital–Weill Cornell Medical Center (academic campus), the vaccine was on-site at the faculty outpatient site on October 23, 2023, most other privately insured sites on November 1, 2023, and the Medicaid-insured clinic on November 17, 2023. At New York Presbyterian–Lower Manhattan Hospital (community-based campus), the RSVpreF vaccine was on-site at most affiliated outpatient sites in a stepwise fashion from November 1 through December 10, 2023. Prior to on-site vaccine availability, the clinical workflow at both hospitals was to electronically prescribe (using the EHR) the vaccine to the patient’s desired pharmacy. The earliest date that a vaccine was administered in our cohort was September 27, 2023, and it was identified via the prescription method.

The primary outcome was PTB, defined as any birth occurring at less than 37 weeks’ gestation. We determined whether the PTB was spontaneous (birth after preterm labor or preterm premature rupture of membranes) or nonspontaneous using available labor and birth data in the repository (eMethods in Supplement 1). Secondary pregnancy outcomes were hypertensive disorders of pregnancy (HDP), small-for–gestational age (SGA) birth weight, and stillbirth. Secondary neonatal outcomes were neonatal intensive care unit (NICU) admission, respiratory distress with NICU admission, jaundice or hyperbilirubinemia, hypoglycemia, and sepsis. HDP was inclusive of gestational hypertension; preeclampsia; eclampsia; and hemolysis, elevated liver enzymes, low platelets (HELLP) syndrome. The diagnoses of HDP, neonatal respiratory distress, neonatal jaundice or hyperbilirubinemia, neonatal hypoglycemia, and neonatal sepsis were based on *ICD-10-CM* codes and cross-checked for data quality with other data available in the repository (eTable 1 in Supplement 1). Stillbirth was based on *ICD-10-CM* codes (eTable 1 in Supplement 1) and total Apgar score of 0 at 1 minute of life. SGA was defined as birth weight in less than the 10th percentile and based on gestational age at birth and neonatal sex using established thresholds.^20,21^ Aside from birth weight, all neonatal outcomes were assessed for occurrence within 5 days of life. Only cases of neonatal respiratory distress that occurred with NICU admission were included to avoid mild cases requiring only initial resuscitation.

### Statistical Analysis

Descriptive statistics were performed using median and IQR for continuous variables, given the nonparametric distributions, and using frequency and percentage for categorical variables. To evaluate whether baseline characteristics were associated with vaccine exposure, bivariate analyses were performed using Wilcoxon rank sum tests for continuous variables and χ^2^ and Fisher exact tests, as appropriate, for categorical variables. Complete case analyses were performed. The false-discovery rate method was used to account for multiple comparisons, and an analog *q* value of .05 was set as the threshold for statistical significance.

We used 3 stepwise approaches to evaluate associations between vaccination and pregnancy outcomes (PTB, HDP, and SGA). First, we ignored all potential biases (naive approach) and used logistic regression models to estimate the associations. Second, multivariable analyses were performed, and covariates were chosen based on whether they were statistically significant (*q* < .05) in bivariate analyses or thought to be potential clinical confounders. Third, in addition to step 2, we accounted for time-dependent vaccine exposure within pregnancy (immortal time bias) using time-dependent covariate Cox regression models. Measures of association are presented with 95% CIs. For the stillbirth outcome, adjusted analyses were not performed given the small number of cases.

Two stratified analyses were performed for the pregnancy outcomes (PTB, HDP, and SGA). The first was based on insurance type (Medicaid/Medicare vs private) and the second on hospital site (Weill Cornell Medical Center vs Lower Manhattan Hospital).

For neonatal outcomes, logistic regression models were performed, and odds ratios (ORs) and 95% CIs were calculated. Stratified analyses were performed based on gestational age at birth (<35 vs ≥35 weeks’ gestation) since this is the institutional threshold for automatic NICU admission and could have affected neonatal outcomes.

Two sensitivity analyses were performed to determine the robustness of our results. First, we excluded all patients who had fewer than 2 prenatal care visit encounters at hospital-affiliated clinical sites, as they likely did not have adequate opportunity to be exposed to vaccination or have documentation of vaccination prenatally. Second, we excluded patients who had an EDD after the study period end (January 31, 2024) to ensure complete gestations for the assessment of pregnancy outcomes.

Given our sample size, a post hoc power analysis suggested that the study had 80% power to detect an absolute risk reduction of 2.6% or greater in the primary outcome of PTB, using a Fisher exact test with a .05 2-sided significance level (assuming a baseline PTB proportion of 6.7% in the nonvaccinated group). R software version 4.2.3 (R Foundation for Statistical Computing) was used for statistical analyses.

## Results

Between September 22, 2023, and January 31, 2024, 2973 eligible pregnant individuals (median [IQR] age, 34.9 [32.4-37.7] years, 618 [20.8%] Asian; 194 [6.5%] Black or African American; 295 [9.9%] Hispanic, Latino, or Spanish origin; 1687 [56.7%] White; and 248 (8.3%) with other race and ethnicity, including American Indian or Alaska Native and Native Hawaiian or Other Pacific Islander) were included after excluding 53 patients for unknown gestational age and 42 for multifetal gestations. These patients delivered at our 2 hospitals at 32 0/7 weeks’ gestation or later. Among them, 1026 (34.5%) had EHR evidence of RSVpreF vaccination before delivery, and 1947 (65.5%) did not. The mean (SD) gestational age at time of vaccination was 34.5 (1.4) weeks. The frequency of vaccination increased steadily, with the steepest increase after the availability of on-site vaccination (Figure).

**Figure.**
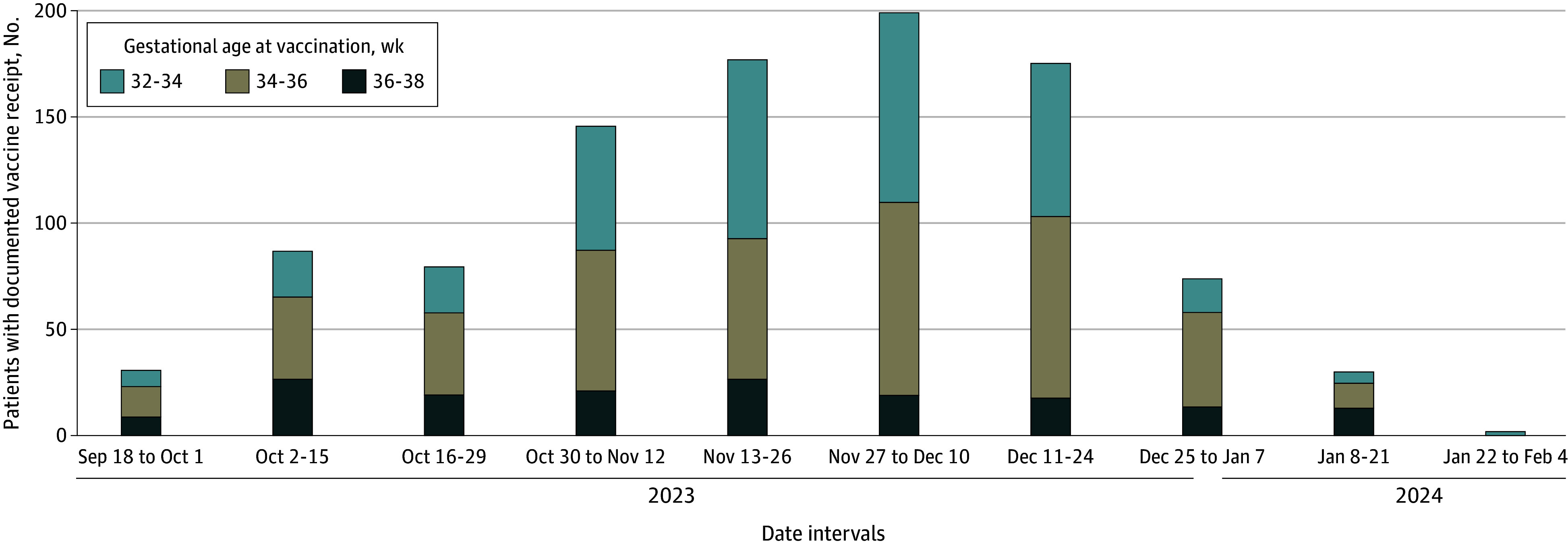
Documented Receipt of Respiratory Syncytial Virus Vaccination During Pregnancy in 2-Week Intervals From September 2023 to January 2024 at 2 Hospitals Within 1 Health System in New York City

The baseline characteristics of patients with and without EHR evidence of prenatal RSVpreF vaccination are shown in Table 1. The vaccinated group on average was older and more likely to be nulliparous, have private insurance, and have a pregnancy by in vitro fertilization. The vaccinated group was less likely to have a diagnosis of pregestational diabetes and an admission body mass index (calculated as weight in kilograms divided by height in meters squared) of 30 or greater. There were significant differences in the distribution of self-identified race and ethnicity and delivery hospital site between groups.

**Table 1.** Characteristics of Patients Who Had RSV Vaccination During Pregnancy Documented in Their Electronic Health Record vs Those Who Did Not

Characteristic	Patients, No. (%)		*P* value	*q* Value
	RSV vaccine (n = 1011)	No RSV vaccine (n = 1962)		
Age, median (IQR), y	35.3 (33.1-38.1)	34.6 (31.9-37.3)	<.001	<.001
Self-identified race^a^				
Asian	201 (19.9)	417 (21.3)	<.001	<.001
Black or African American	44 (4.4)	150 (7.6)		
White	618 (61.1)	1069 (54.5)		
Other^b^	65 (6.4)	183 (9.3)		
Multiple races^c^	28 (2.8)	49 (2.5)		
Declined	55 (5.4)	94 (4.8)		
Self-identified ethnicity^a^				
Hispanic or Latino or Spanish origin	73 (7.2)	222 (11.3)	.001	.002
Not Hispanic or Latino or Spanish origin	866 (85.7)	1632 (82.9)		
Declined	72 (7.1)	114 (5.8)		
Insurance type				
Medicaid or Medicare	60 (5.9)	393 (20.0)	<.001	<.001
Private	950 (94.0)	1566 (79.9)		
Other or unknown	1 (0.1)	3 (0.1)		
Former or current smoker	86 (8.7)	143 (8.0)	.50	.60
Illicit drug use during pregnancy^d^	16 (1.9)	25 (1.6)	.60	.70
BMI ≥30 at delivery admission^e^	316 (31.4)	666 (34.7)	.07	.11
Chronic hypertension	7 (0.7)	7 (0.4)	.30	.40
Asthma	103 (10.2)	212 (10.8)	.60	.70
Systemic lupus erythematosus	2 (0.2)	7 (0.4)	.70	.80
Inflammatory bowel disease	42 (4.2)	95 (4.8)	.40	.50
Pregestational diabetes	7 (0.7)	30 (1.5)	.05	.09
Gestational diabetes	55 (5.4)	119 (6.1)	.50	.60
In vitro fertilization pregnancy	113 (11.2)	141 (7.2)	<.001	<.001
Nulliparous	623 (61.6)	1128 (57.5)	.03	.06
Delivery hospital site				
Weill Cornell Medical Center	781 (77.3)	1646 (83.9)	<.001	<.001
Lower Manhattan Hospital	230 (22.7)	316 (16.1)		
Cesarean delivery	329 (32.5)	639 (32.6)	>.99	>.99
Infant sex				
Female	497 (49.2)	931(47.5)	.40	.50
Male	514 (50.8)	1031 (52.5)		

Abbreviations: BMI, body mass index (calculated as weight in kilograms divided by height in meters squared); RSV, respiratory syncytial virus.

^a^ Racial and ethnic group were based on documentation in the electronic health record, which is typically captured by self-report during visit encounters.

^b^ Inclusive of American Indian or Alaskan Native and Native Hawaiian or Other Pacific Islander.

^c^ Indicates a specific category called “Other combinations not described” in the electronic health record.

^d^ Data are available for 854 patients who had evidence of vaccination and 1564 patients who did not have evidence of vaccination.

^e^ Data are available for 1007 patients who had evidence of vaccination and 1918 patients who did not have evidence of vaccination.

Overall PTB occurred in 191 of 2973 patients (6.5%) in the cohort. EHR evidence of RSVpreF vaccination during pregnancy was not significantly associated with an increased risk for PTB (OR, 0.88; 95% CI, 0.64-1.20) (Table 2). This finding remained unchanged in multivariable analyses (aOR, 0.87; 95% CI, 0.62-1.20) and the time-dependent covariate Cox regression model (hazard ratio [HR], 0.93; 95% CI, 0.64-1.34). In both groups, there were more spontaneous PTB cases than nonspontaneous PTB cases: 38 of 60 (63.3%) spontaneous and 22 (36.7%) nonspontaneous in the vaccinated group vs 69 of 131 (52.7%) spontaneous and 62 (47.3%) in the unvaccinated group. There were no significant differences in SGA or stillbirth, but there was an increased risk of overall HDP with RSVpreF vaccination in the time-dependent model (HR, 1.43; 95% CI, 1.16-1.77) (Table 2). In stratified analyses, there were differences noted for risks of HDP and SGA based on insurance type and hospital site (eTables 2-4 in Supplement 1). Sensitivity analyses did not reveal additional findings (eTables 5 and 6 in Supplement 1).

**Table 2.** Pregnancy Outcomes Between Patients Who Had RSV Vaccination During Pregnancy Documented in Their Electronic Health Record vs Those Who Did Not

Pregnancy outcome	Patients, No. (%)		OR (95% CI)	aOR (95% CI)^a^	HR (95% CI)^b^
	RSV vaccine (n = 1011)	No RSV vaccine (n = 1962)			
Primary outcome					
Preterm birth <37 weeks’ gestation	60 (5.9)	131 (6.7)	0.88 (0.64-1.20)	0.87 (0.62-1.20)	0.93 (0.64-1.34)
Secondary outcomes					
Hypertensive disorders of pregnancy	203 (20.1)	355 (18.1)	1.14 (0.94-1.38)	1.10 (0.90-1.35)	1.43 (1.16-1.77)
Gestational hypertension^c^	153 (15.1)	273 (13.9)	NA	NA	NA
Preeclampsia	67 (6.6)	130 (6.6)	NA	NA	NA
Eclampsia	1 (0.1)	1 (0.1)	NA	NA	NA
HELLP syndrome	2 (0.2)	2 (0.1)	NA	NA	NA
Small-for–gestational age birth weight^d^	107 (10.6)	178 (9.1)	1.19 (0.92-1.52)	1.16 (0.89-1.50)	1.31 (0.97-1.77)
Stillbirth	2 (0.2)	3 (0.2)	1.29 (0.17-7.82)	NA	NA

Abbreviations: aOR, adjusted odds ratio; HELLP, hemolysis, elevated liver enzymes, and low platelets; HR, hazard ratio; NA, not applicable; OR, odds ratio; RSV, respiratory syncytial virus.

^a^ Multivariable logistic regression model including covariates maternal age, race, ethnicity, insurance type, parity, delivery hospital site, in vitro fertilization pregnancy, pregestational diabetes, and body mass index (calculated as weight in kilograms divided by height in meters squared) greater than 30 at delivery encounter admission.

^b^ Time-dependent Cox covariate regression model including same covariates as multivariable logistic regression model.

^c^ The denominator removes patients with diagnosis of preexisting chronic hypertension, 7 patients in each study group.

^d^ Small for gestational age determined based on gestational age (in weeks) at birth and sex using the Fenton reference.^16,17^

Among offspring, there were no significant differences in outcomes (Table 3). In the stratified analyses based on less than 35 weeks’ gestation vs 35 weeks’ gestation or longer at birth (eTable 7 in Supplement 1) and sensitivity analyses (eTables 5 and 6 in Supplement 1), we found similar nonsignificant findings.

**Table 3.** Secondary Neonatal Outcomes Between Offspring Born to Patients Who Had Documentation of Receiving RSV Vaccine During Pregnancy vs Those Born to Patients Who Did Not

Neonatal outcome	Patients, No. (%)		OR (95% CI)
	RSV vaccine (n = 1011)	No RSV vaccine (n = 1962)	
Admission to the NICU	89 (8.8)	159 (8.1)	1.09 (0.83-1.43)
Respiratory distress with NICU admission	56 (5.5)	95 (4.8)	1.15 (0.82-1.61)
Jaundice or hyperbilirubinemia	191 (18.1)	362 (18.5)	1.03 (0.85-1.25)
Hypoglycemia	59 (5.8)	133 (6.8)	0.85 (0.62-1.16)
Sepsis	3 (0.3)	9 (0.5)	0.65 (0.14-2.17)

Abbreviations: NICU, neonatal intensive care unit; OR, odds ratio; RSV, respiratory syncytial virus.

## Discussion

At our 2 hospitals, 34.5% of individuals who delivered during the 2023 to 2024 RSV vaccination season had documented evidence of prenatal RSVpreF vaccination. RSVpreF vaccination steadily increased throughout the 2023 to 2024 RSV season, with the largest increase seen after on-site vaccine availability. In the main analyses, there were no significant differences in maternal or perinatal outcomes between patients who had EHR evidence of prenatal RSVpreF vaccination and those who did not. However, there were increased risks of HDP and SGA birth weight seen when accounting for immortal time bias, hospital site, and insurance type.

The vaccine uptake among our patients is higher than what has been reported nationally. In contrast to our vaccination frequency of 34.5%, the CDC reports the overall coverage with the RSV vaccine nationally was 17.8% during the same time period.^22^ While the sociodemographic profile of our patient population (majority self-identified as non-Hispanic, White, or Asian with private insurance) may reflect people more likely to receive vaccination, we believe that our hospitals’ early efforts to optimize equitable vaccination access by stocking and administering the vaccine in most clinical sites promoted vaccination among our patients. Indeed, receipt of RSVpreF vaccine increased most substantially after on-site vaccine availability.

The CDC report showed that RSVpreF vaccination coverage was highest among non-Hispanic Asian (24.8%) and lowest among non-Hispanic Black (10.3%) pregnant individuals.^22^ Our study similarly found significant differences in the distribution of self-identified race and ethnicity based on vaccination status. We found a significantly lower vaccination frequency among patients who self-identified as Black or Hispanic and those with government insurance. It is well recognized that for other recommended prenatal vaccines such influenza, COVID-19, and tetanus-diphtheria-pertussis, vaccination rates are significantly lower in non-Hispanic Black or African American and Hispanic or Latina pregnant individuals compared with their non-Hispanic White counterparts.^23^ Further investigation is needed to examine RSVpreF vaccination barriers, such as general vaccine hesitancy during pregnancy, lack of access, cost, and patient preference for nirsevimab for their infant.

We did not find a significant difference in overall PTB at less than 37 weeks’ gestation between our study groups. In contrast to the trials,^4^ which observed more cases of PTB at less than 37 weeks’ gestation among RSVpreF vaccine recipients than among placebo recipients (although not statistically significant), we observed fewer cases of PTB among our patients with EHR evidence of vaccination compared with those without. It is possible that we found a different trend because our study was performed in the postmarket period when the vaccine was recommended to be administered in the window of 32 0/7 to 36 6/7 weeks’ gestation (compared with the 24 0/7 to 36 6/7 weeks’ gestation window used in the trials^4^). However, it is notable that, even in the trial,^4^ the trend was driven by imbalances in PTB in low- and middle-income countries. In their analysis of US births only, the PTB rate was 5.1% (126 of 2494) in the vaccine group compared with 5.1% (126 of 2484) in the control group.^4^ Additionally, the original analysis^4^ of US participants enrolled during the approved dosing interval (32 0/7 to 36 6/7 weeks’ gestation) demonstrated that the imbalance was reversed, with PTBs occurring in 4.0% (721 of 1628) in the vaccine group compared with 4.4% (732 of 1604) in the control group. Our study shows a similarly reassuring trend for PTB. While our overall frequency of PTB is higher than the trial, this is likely because we did not limit our cohort to healthy individuals, as was done in the trial^4^ (ie, exclusion of patients with endocrine disorders, in vitro fertilization, and prior or current pregnancy complications).

In the time-dependent model only, we found a significantly increased risk of overall HDP in the vaccinated group. The trial^4^ also observed more cases of gestational hypertension and preeclampsia among RSVpreF vaccine recipients than among placebo recipients, although these associations were not statistically significant. In our stratified analyses, the significantly increased risks of HDP appeared to be associated with insurance type and hospital site. These findings should be investigated in other populations and settings. In our main analyses, we did not find significant differences overall in SGA, stillbirth, or other neonatal outcomes, such as NICU admission, respiratory distress, hypoglycemia, jaundice or hyperbilirubinemia, or sepsis, between the 2 groups.

### Strengths and Limitations

Our study has several strengths. First, it provides clinical data for the first RSV season in the continental US during the postmarket period of an FDA-approved and nationally recommended RSV vaccine specific to the pregnant population. Second, we had a higher proportion of vaccinated patients compared with nationally reported estimates,^22^ which allowed us to make important comparisons. Third, we included most patients who delivered at our 2 hospitals, which expands on the trial population,^4^ which included only patients with low risk. Fourth, we explored important neonatal outcomes not previously reported in the trial.^4^ Fifth, we utilized several statistical approaches to minimize potential biases that commonly affect postmarket observational vaccine studies.^24,25^

There are notable limitations. First, our patient population and our NYC location may not be generalizable to other settings. Second, RSVpreF vaccination status was based on EHR documentation. Although we utilized multiple channels to comprehensively capture immunization data, we may not have captured vaccinations performed at smaller non-PBM–owned pharmacies or clinical sites not part of the EHR. Therefore, it is possible that some patients were misclassified as not having received vaccination when they in fact did, potentially biasing results toward the null. Third, there may be a residual risk of immortal time bias^26^ for outcomes like PTB, since the modified vaccination window recommended made it more likely to be administered at gestations near full term (ie, close to 37 weeks’ gestation). Fourth, several outcome variables, including HDP, neonatal respiratory distress, neonatal hypoglycemia, and neonatal hyperbilirubinemia or jaundice, were based on *ICD-10-CM* codes and not individually confirmed. However, we sought to maximize data validity by cross-checking them with clinical data. Fifth, we had a higher vaccination frequency than what is nationally estimated,^22^ and our PTB risk trended opposite to what was previously suggested; however, our sample size may still be underpowered, and the risk of type II error persists.

## Conclusions

In this cohort study of pregnant individuals who delivered at 32 weeks of gestation or later at 2 NYC hospitals, EHR evidence of prenatal RSVpreF vaccination was not associated with PTB. These data add to the existing evidence supporting the overall safety of prenatal RSVpreF vaccination. However, there may be increased risks of HDP and SGA when addressing immortal time bias, hospital site, and insurance type, and these associations should be investigated further. Additionally, prenatal RSVpreF vaccination remains underutilized, and exploration of the factors and disparities associated with prenatal vaccination is needed.

## Notes

Supplement 1. **eMethods.** Hierarchical Logic for Determination of Spontaneous vs Nonspontaneous Indication for Delivery Based on Available Labor and Delivery Data in Repository **eTable 1.** *International Classification of Diseases, Tenth Revision, Clinical Modification* (*ICD-10-CM*) Codes Used for Data Variables **eTable 2.** Stratified Analyses for the Association Between Respiratory Syncytial Virus Vaccination and the Risks of Preterm Birth at Less Than 37 Weeks of Gestation by Maternal Insurance Type and Delivery Hospital Site **eTable 3.** Stratified Analyses for the Association Between Respiratory Syncytial Virus Vaccination and the Risks of Hypertensive Disorders of Pregnancy by Maternal Insurance Type and Delivery Hospital Site **eTable 4.** Stratified Analyses for the Association Between Respiratory Syncytial Virus Vaccination and the Risks of Small for Gestational Age Birthweight by Maternal Insurance Type and Delivery Hospital Site **eTable 5.** Sensitivity Analyses for the Association Between Maternal Respiratory Syncytial Virus Vaccination and Pregnancy Outcomes Among Mother-Infant Dyads with Evidence of Prenatal Care at Hospital-Affiliated Clinics During Pregnancy **eTable 6.** Sensitivity Analyses for the Association Between Maternal Respiratory Syncytial Virus Vaccination and Pregnancy Outcomes Among Patients with Estimated Due Dates On or Before January 31, 2024 **eTable 7.** Stratified Analyses for the Association Between Respiratory Syncytial Virus Vaccination and the Risks of Adverse Neonatal Outcomes by Gestational Age at Birth

Supplement 2.
Data Sharing Statement
