# Supplementary material for: Nonadjuvanted Bivalent Respiratory Syncytial Virus Vaccination and Perinatal Outcomes
Source: JAMA Netw Open. 2024 Jul 8;7(7):e2419268. doi: 10.1001/jamanetworkopen.2024.19268 (PMC11231799; doi:10.1001/jamanetworkopen.2024.19268)
Supplement: Supplement 1. — eMethods. Hierarchical Logic for Determination of Spontaneous vs Nonspontaneous Indication for Delivery Based on Available Labor and Delivery Data in Repository eTable 1. International Classification of Diseases, Tenth Revision, Clinical Modification (ICD-10-CM) Codes Used for Data Variables eTable 2. Stratified Analyses for the Association Between Respiratory Syncytial Virus Vaccination and the Risks of Preterm Birth at Less Than 37 Weeks of Gestation by Maternal Insurance Type and Delivery Hospital Site eTable 3. Stratified Analyses for the Association Between Respiratory Syncytial Virus Vaccination and the Risks of Hypertensive Disorders of Pregnancy by Maternal Insurance Type and Delivery Hospital Site eTable 4. Stratified Analyses for the Association Between Respiratory Syncytial Virus Vaccination and the Risks of Small for Gestational Age Birthweight by Maternal Insurance Type and Delivery Hospital Site eTable 5. Sensitivity Analyses for the Association Between Maternal Respiratory Syncytial Virus Vaccination and Pregnancy Outcomes Among Mother-Infant Dyads with Evidence of Prenatal Care at Hospital-Affiliated Clinics During Pregnancy eTable 6. Sensitivity Analyses for the Association Between Maternal Respiratory Syncytial Virus Vaccination and Pregnancy Outcomes Among Patients with Estimated Due Dates On or Before January 31, 2024 eTable 7. Stratified Analyses for the Association Between Respiratory Syncytial Virus Vaccination and the Risks of Adverse Neonatal Outcomes by Gestational Age at Birth [file jamanetwopen-e2419268-s001.pdf]

Supplemental Online Content

Son M, Riley LE, Staniczenko AP, et al. Nonadjuvanted bivalent respiratory syncytial virus vaccination and perinatal outcomes. *JAMA Netw Open.* 2024;7(7):e2419268. doi:10.1001/jamanetworkopen.2024.19268

eMethods. Hierarchical Logic for Determination of Spontaneous vs Nonspontaneous Indication for Delivery Based on Available Labor and Delivery Data in Repository

eTable 1. *International Classification of Diseases, Tenth Revision, Clinical Modification* (*ICD-10-CM*) Codes Used for Data Variables

eTable 2. Stratified Analyses for the Association Between Respiratory Syncytial Virus Vaccination and the Risks of Preterm Birth at Less Than 37 Weeks of Gestation by Maternal Insurance Type and Delivery Hospital Site

eTable 3. Stratified Analyses for the Association Between Respiratory Syncytial Virus Vaccination and the Risks of Hypertensive Disorders of Pregnancy by Maternal Insurance Type and Delivery Hospital Site

eTable 4. Stratified Analyses for the Association Between Respiratory Syncytial Virus Vaccination and the Risks of Small for Gestational Age Birthweight by Maternal Insurance Type and Delivery Hospital Site

eTable 5. Sensitivity Analyses for the Association Between Maternal Respiratory Syncytial Virus Vaccination and Pregnancy Outcomes Among Mother-Infant Dyads with Evidence of Prenatal Care at Hospital-Affiliated Clinics During Pregnancy

eTable 6. Sensitivity Analyses for the Association Between Maternal Respiratory Syncytial Virus Vaccination and Pregnancy Outcomes Among Patients with Estimated Due Dates On or Before January 31, 2024

eTable 7. Stratified Analyses for the Association Between Respiratory Syncytial Virus Vaccination and the Risks of Adverse Neonatal Outcomes by Gestational Age at Birth

This supplemental material has been provided by the authors to give readers additional information about their work.

© 2024 Son M et al. *JAMA Network Open.*

eMethods. Hierarchical Logic for Determination of Spontaneous vs Nonspontaneous Indication for Delivery Based on Available Labor and Delivery Data in Repository

For Final_Spontaneous_YN, the hierarchal logic is:

1. Spontaneous_YN
2. C-Section Spontaneous_YN

Hierarchal logic for Spontaneous_YN:

1. If Labor onset < admission time THEN 1
2. If Rupture type is either Premature or Spontaneous THEN 1
3. If Rupture type is either Artificial, Intact, Bulging THEN 0
4. If Induction is not null/not none THEN 0
5. If Augmentation is not null/not none, THEN 1
6. If Induction and Augmentation are none THEN 1
7. The rest are Unknowns (basically Labor onset < admission AND (Rupture type is null or none) AND (Augmentation is null or none))

Hierarchal logic for C_Section_Spontaneous_YN:

1. If Arrest of Labor 1st Stage or Arrest of Labor 2nd stage is indicated THEN 1
2. If [Abnormal Placentation],[Breech],[Failed Induction],[Known/Suspected Fetal Anomaly],[Malposition],[Malpresentation],[Maternal Infection (HSV/HIV)],[Maternal Request],[Multiple Gestation],[Prior cesarean],[Prior Non-Cesarean Uterine Surgery],[Suspected Macrosomia], or [Unstable Lie] is indicated THEN 0
3. If NULL, category II tracing, cord prolapse, fetal intolerance of labor, placental abruption, category III tracing, or failed vacuum is indicated THEN Unknown

Overall hierarchal logic

1. If Spontaneous_YN = 1 THEN Final_spontaneous_YN = 1
2. If Spontaneous_YN = 0 and C_Sect_Spontaneous_YN = 1 THEN Final_spontaneous = 1
3. If Spontaneous_YN = Unknown and C_Sect_Spontaneous_YN != Uknown THEN Final_spontaneous = C_sect_spontaneous (this might either be 1 or 0)
4. Rest is Final_spontaneous = Spontaneous_YN

© 2024 Son M et al. *JAMA Network Open.*

eTable 1. ***International Classification of Diseases, Tenth Revision, Clinical Modification* (*ICD-10-CM*) Codes Used for Data Variables**

**Data variable**	**ICD-10-CM codes**
Asthma	J45.2; J45.20; J45.21; J45.22; J45.3; J45.30; J45.31; J45.32; J45.4; J45.40; J45.41; J45.42; J45.5; J45.50; J45.51; J45.52; J45.90; J45.901; J45.902; J45.909
Chronic hypertension	I11.0; I11.9; I12; I12.0; I12.9; I13; I13.0; I13.1; I13.2; I15; I15.0; I15.1; I15.2; I15.8; I15.9; I16; I16.0; I16.1; I16.9
Eclampsia[a]	O15; O15.0; O15.00; O15.02; O15.03; O15.1; O15.2; O15.9
Gestational diabetes mellitus	O24.4; O24.41; O24.410; O24.414; O24.415; O24.419; O24.42; O24.420; O24.424; O24.425; O24.429; O24.43; O24.430; O24.434; O24.435; O24.439
Gestational hypertension[a]	O12; O12.0; O12.00; O12.01; O12.02; O12.03; O12.04; O12.05; O12.1; O12.10; O12.11; O12.12; O12.13; O12.14; O12.15; O12.2; O12.20; O12.21; O12.22; O12.23; O12.24; O12.25; O13; O13.1; O13.2; O13.3; O13.4; O13.5; O13.9
Hemolysis, elevated liver enzymes, and low platelet (HELLP) syndrome[a]	O14.2; O14.20; O14.22; O14.23; O14.24; O14.25
In vitro fertilization pregnancy	O09.81; O09.811; O09.812; O09.813; O09.819
Inflammatory bowel disease	K50.90; K51.20; K51.90; K51.912; K51.913; K51.914; K51.919; O99.611; O99.612; O99.613; O99.619
Neonatal hypoglycemia[b]	E16.1; E16.2; P70.3; P70.4
Neonatal jaundice or hyperbilirubinemia[c]	P58; P58.1; P58.2; P58.3; P58.4; P58.41; P58.42; P58.8; P58.9; P59; P59.1; P59.2; P59.20; P59.29; P59.3; P59.8; P59.9; R17
Neonatal respiratory distress	P22; P22.0; P22.1; P22.8; P22.9; P24.0; P24.00; P24.01; P27.1
Neonatal sepsis	P36.1; P36.2; P36.3; P36.4; P36.5; P36.8; P36.9
Preeclampsia[a]	O14; O14.0; O14.00; O14.02; O14.03; O14.04; O14.05; O14.1; O14.10; O14.12; O14.13; O14.14; O14.15; O14.9; O14.90; O14.92; O14.93; O14.94; O14.95
Pre-gestational diabetes mellitus	E10; E10.1; E10.10; E10.11; E10.2; E10.21; E10.22; E10.29; E10.3; E10.31; E10.311; E10.319; E10.32; E10.321; E10.3211; E10.3212; E10.3213; E10.3219; E10.329; E10.3291; E10.3292; E10.3293; E10.3299; E10.33; E10.331; E10.3311; E10.3312; E10.3313; E10.3319; E10.339; E10.3391; E10.3392; E10.3393; E10.3399; E10.34; E10.341; E10.3411; E10.3412; E10.3413; E10.3419; E10.349; E10.3491; E10.3492; E10.3493; E10.3499; E10.35; E10.351; E10.3511; E10.3512; E10.3513; E10.3519; E10.352; E10.3521; E10.3522; E10.3523; E10.3529; E10.353; E10.3531; E10.3532;

© 2024 Son M et al. *JAMA Network Open.*

Data variable	ICD-10-CM codes
Pre-gestational diabetes mellitus, continued	E10.3533; E10.3539; E10.354; E10.3541; E10.3542; E10.3543; E10.3549; E10.355; E10.3551; E10.3552; E10.3553; E10.3559; E10.359; E10.3591; E10.3592; E10.3593; E10.3599; E10.36; E10.37; E10.37X1; E10.37X2; E10.37X3; E10.37X9; E10.39; E10.4; E10.40; E10.41; E10.42; E10.43; E10.44; E10.49;E10.5; E10.51; E10.52; E10.59; E10.6; E10.61; E10.610; E10.618; E10.62; E10.620; E10.621; E10.622; E10.628; E10.63; E10.630; E10.638; E10.64; E10.640; E10.641; E10.649; E10.65; E10.69; E10.8; E10.9; E11; E11.0; E11.00; E11.01; E11.1; E11.10; E11.11; E11.2; E11.21; E11.22; E11.29; E11.3; E11.31; E11.311; E11.319; E11.32; E11.321; E11.3211; E11.3212; E11.3213; E11.3219; E11.329; E11.3291; E11.3292; E11.3293; E11.3299; E11.33; E11.331; E11.3311; E11.3312; E11.3313; E11.3319; E11.339; E11.3391; E11.3392; E11.3393; E11.3399; E11.34; E11.341; E11.3411; E11.3412; E11.3413; E11.3419; E11.349; E11.3491; E11.3492; E11.3493; E11.3499; E11.35; E11.351; E11.3511; E11.3512; E11.3513; E11.3519; E11.352; E11.3521; E11.3522; E11.3523; E11.3529; E11.353; E11.3531; E11.3532; E11.3533; E11.3539; E11.354; E11.3541; E11.3542; E11.3543; E11.3549; E11.355; E11.3551; E11.3552; E11.3553; E11.3559; E11.359; E11.3591; E11.3592; E11.3593; E11.3599; E11.36; E11.37; E11.37X1; E11.37X2; E11.37X3; E11.37X9; E11.39; E11.4; E11.40; E11.41; E11.42; E11.43; E11.44; E11.49; E11.5; E11.51; E11.52; E11.59; E11.6; E11.61; E11.610; E11.618; E11.62; E11.620; E11.621; E11.622; E11.628; E11.63; E11.630; E11.638; E11.64; E11.640; E11.641; E11.649; E11.65; E11.69; E11.8; E11.9
Stillbirth	P95; Z37.1; Z37.9; IMO0002
Systemic lupus erythematosus	L93; L93.0; L93.1; L93.2; M32; M32.0; M32.1; M32.10; M32.11; M32.12; M32.13; M32.14; M32.15; M32.19; M32.8; M32.9

[a] Cases with these ICD-10-CM codes were cross-checked for the presence of ≥ two elevated blood pressure values (≥140 systolic or ≥90 diastolic) during the delivery hospitalization encounter as a data quality check to support these diagnoses.

[b] The lowest recorded glucose level was examined for each neonate, and those who had these ICD-10-CM codes were cross-checked with those who had a lowest glucose level <45 mg/dL as a data quality check.

[c] The highest recorded bilirubin level was examined for each neonate, and those who had these ICD-10-CM codes were cross-checked with those who had a highest bilirubin level >10 mg/dL.

© 2024 Son M et al. *JAMA Network Open.*

eTable 2. Stratified Analyses for the Association Between Respiratory Syncytial Virus Vaccination and the Risks of Preterm Birth at Less Than 37 Weeks of Gestation by Maternal Insurance Type and Delivery Hospital Site

	Event (%)	Unadjusted RR (95% CI)	Adjusted Analyses	
			aRR (95% CI)[a]	HR (95% CI)[b]
**Medicaid/Medicare**				
No EHR evidence of RSV vaccination	31/393 (7.9)	Reference	Reference	Reference
EHR evidence of RSV vaccination	5/60 (8.3)	1.06 (0.35-2.63)	1.08 (0.34-2.84)	1.30 (0.44-3.87)
**Private Insurance**				
No EHR evidence of RSV vaccination	99/1566 (6.3)	Reference	Reference	Reference
EHR evidence of RSV vaccination	54/950 (5.7)	0.89 (0.63-1.25)	0.86 (0.60-1.21)	0.86 (0.58-1.29)
**Weill Cornell Medical Center**				
No EHR evidence of RSV vaccination	109/1646 (6.6)	Reference	Reference	Reference
EHR evidence of RSV vaccination	48/781 (6.1)	0.92 (0.65-1.30)	0.90 (0.62-1.29)	0.98 (0.64-1.50)
**Lower Manhattan Hospital**				
No EHR evidence of RSV vaccination	22/316 (7.0)	Reference	Reference	Reference
EHR evidence of RSV vaccination	12/230 (5.2)	0.74 (0.35-1.74)	0.80 (0.35-1.74)	0.78 (0.33-1.83)

[a] Multivariable logistic regression model including covariates maternal age, race, ethnicity, insurance type, parity, delivery hospital site, in vitro fertilization pregnancy, pre-gestational diabetes mellitus, and body mass index ≥30 kg/m^2 at delivery encounter admission.
[b] Time-dependent Cox covariate regression model including same covariates.

© 2024 Son M et al. *JAMA Network Open.*

eTable 3. Stratified Analyses for the Association Between Respiratory Syncytial Virus Vaccination and the Risks of Hypertensive Disorders of Pregnancy by Maternal Insurance Type and Delivery Hospital Site

	Event (%)	Unadjusted RR (95% CI)	Adjusted Analyses	
			aRR (95% CI)[a]	HR (95% CI)[b]
**Medicaid/Medicare**				
No EHR evidence of RSV vaccination	78/393 (20.0)	Reference	Reference	Reference
EHR evidence of RSV vaccination	17/60 (28.0)	1.60 (0.85-2.91)	1.43 (0.72-2.75)	1.24 (0.66-2.34)
**Private Insurance**				
No EHR evidence of RSV vaccination	277/1566 (18.0)	Reference	Reference	Reference
EHR evidence of RSV vaccination	185/950 (19.0)	1.13 (0.91-1.38)	1.07 (0.87-1.33)	1.53 (1.21-1.93)
**Weill Cornell Medical Center**				
No EHR evidence of RSV vaccination	295/1646 (18.0)	Reference	Reference	Reference
EHR evidence of RSV vaccination	159/781 (20.0)	1.17 (0.94-1.45)	1.14 (0.91-1.43)	1.43 (1.12-1.83)
**Lower Manhattan Hospital**				
No EHR evidence of RSV vaccination	60/316 (19.0)	Reference	Reference	Reference
EHR evidence of RSV vaccination	44/230 (19.0)	1.01 (0.65-1.55)	0.93 (0.57-1.49)	1.59 (0.99-2.54)

[a]Multivariable logistic regression model including covariates maternal age, race, ethnicity, insurance type, parity, delivery hospital site, in vitro fertilization pregnancy, pre-gestational diabetes mellitus, and body mass index ≥30 kg/m^2 at delivery encounter admission.
[b]Time-dependent Cox covariate regression model including same covariates.

© 2024 Son M et al. *JAMA Network Open.*

eTable 4. Stratified Analyses for the Association Between Respiratory Syncytial Virus Vaccination and the Risks of Small for Gestational Age Birthweight by Maternal Insurance Type and Delivery Hospital Site

	Event (%)	Unadjusted RR (95% CI)	Adjusted Analyses	
			aRR (95% CI)[a]	HR (95% CI)[b]
**Medicaid/Medicare**				
No EHR evidence of RSV vaccination	37/393 (9.4)	Reference	Reference	Reference
EHR evidence of RSV vaccination	4/60 (6.7)	0.69 (0.20-1.80)	0.61 (0.17-1.70)	0.45 (0.14-1.42)
**Private Insurance**				
No EHR evidence of RSV vaccination	141/1566 (9.0)	Reference	Reference	Reference
EHR evidence of RSV vaccination	103/950 (11.0)	1.23 (0.94-1.60)	1.20 (0.91-1.57)	1.44 (1.04-1.99)
**Weill Cornell Medical Center**				
No EHR evidence of RSV vaccination	149/1646 (9.1)	Reference	Reference	Reference
EHR evidence of RSV vaccination	74/781 (9.5)	1.05 (0.78-1.40)	1.02 (0.75-1.37)	2.54 (1.36-4.74)
**Lower Manhattan Hospital**				
No EHR evidence of RSV vaccination	29/316 (9.2)	Reference	Reference	Reference
EHR evidence of RSV vaccination	33/230 (14.0)	1.66 (0.98-2.83)	1.83 (1.01-3.35)	2.54 (1.36-4.74)

[a]Multivariable logistic regression model including covariates maternal age, race, ethnicity, insurance type, parity, delivery hospital site, in vitro fertilization pregnancy, pre-gestational diabetes mellitus, and body mass index ≥30 kg/m^2 at delivery encounter admission.
[b]Time-dependent Cox covariate regression model including same covariates.

eTable 5. Sensitivity Analyses for the Association Between Maternal Respiratory Syncytial Virus Vaccination and Pregnancy Outcomes Among Mother-Infant Dyads with Evidence of Prenatal Care at Hospital-Affiliated Clinics During Pregnancy

	Patients, No. (%)				
**Outcome**	**RSV vaccine (n=987)**	**No RSV vaccine (n=1732)**	**OR (95% CI)**	**aOR (95% CI)[a]**	**HR (95% CI)[b]**
Primary outcome					
Preterm birth <37 weeks' gestation	59 (6.0)	114 (6.6)	0.90 (0.65-1.24)	0.89 (0.63-1.25)	0.94 (0.65-1.36)
Secondary outcomes					
Hypertensive disorders of pregnancy	203 (20.6)	316 (18.2)	1.16 (0.95-1.41)	1.15 (0.93-1.41)	1.49 (1.20-1.86)
Gestational hypertension	153 (15.5)	250 (14.4)	NA		NA
Preeclampsia	67 (6.8)	110 (6.4)	NA		NA
Eclampsia	1 (0.1)	0 (0.0)	NA		NA
HELLP syndrome	2 (0.2)	1 (0.1)	NA		NA
Small for gestational age birthweight	103 (10.4)	155 (8.9)	1.19 (0.91-1.54)	1.11 (0.84-1.46)	1.28 (0.94-1.74)
Stillbirth	2 (0.2)	3 (0.2)	1.17 (0.15-7.07)	NA	NA
Admission to the NICU	88 (8.9)	139 (8.0)	1.12 (0.85-1.48)	NA	NA
Respiratory distress with NICU admission	55 (5.6)	86 (5.0)	1.13 (0.79-1.59)	NA	NA
Jaundice or hyperbilirubinemia	186 (18.8)	338 (19.5)	0.96 (0.78-1.17)	NA	NA
Hypoglycemia	59 (6.0)	125 (7.2)	0.82 (0.59-1.12)	NA	NA
Sepsis	3 (0.3)	9 (0.5)	0.58 (0.13-1.96)	NA	NA

[a]Multivariable logistic regression model including covariates maternal age, race, ethnicity, insurance type, parity, delivery hospital site, in vitro fertilization pregnancy, pre-gestational diabetes mellitus, and body mass index ≥30 kg/m^2 at delivery encounter admission.

[b]Time-dependent Cox covariate regression model including same covariates.

© 2024 Son M et al. *JAMA Network Open.*

eTable 6. Sensitivity Analyses for the Association Between Maternal Respiratory Syncytial Virus Vaccination and Pregnancy Outcomes Among Patients with Estimated Due Dates On or Before January 31, 2024

	Patients, No. (%)				
**Outcome**	**RSV vaccine (n=862)**	**No RSV vaccine (n=1892)**	**OR (95% CI)**	**aOR (95% CI)[a]**	**HR (95% CI)[b]**
Primary outcome					
Preterm birth <37 weeks' gestation	36 (4.2)	102 (5.4)	0.76 (0.51-1.12)	0.80 (0.53-1.20)	0.94 (0.81-1.12)
Secondary outcomes					
Hypertensive disorders of pregnancy	167 (19.4)	345 (18.2)	1.08 (0.88-1.32)	1.05 (0.84-1.30)	1.49 (1.20-1.86)
Gestational hypertension	130 (15.1)	266 (14.1)	NA	NA	NA
Preeclampsia	54 (6.3)	126 (6.7)	NA	NA	NA
Eclampsia	0 (0.0)	1 (0.1)	NA	NA	NA
HELLP syndrome	2 (0.2)	2 (0.1)	NA	NA	NA
Small for gestational age birthweight	85 (9.9)	175 (9.2)	1.07 (0.81-1.41)	1.06 (0.80-1.41)	1.28 (0.94-1.74)
Stillbirth			1.46 (0.19-8.85)	NA	NA
Admission to the NICU	75 (8.7)	139 (7.4)	1.20 (0.89-1.61)	NA	NA
Respiratory distress with NICU admission	46 (5.3)	83 (4.4)	1.23 (0.84-1.77)	NA	NA
Jaundice or hyperbilirubinemia	154 (17.9)	343 (18.1)	0.98 (0.79-1.21)	NA	NA
Hypoglycemia	42 (4.9)	120 (6.3)	0.76 (0.52-1.08)	NA	NA
Sepsis	3 (0.3)	9 (0.5)	0.73 (0.16-2.46)	NA	NA

[a]Multivariable logistic regression model including covariates maternal age, race, ethnicity, insurance type, parity, delivery hospital site, in vitro fertilization pregnancy, pre-gestational diabetes mellitus, and body mass index ≥30 kg/m^2 at delivery encounter admission.
[b]Time-dependent Cox covariate regression model including same covariates.

eTable 7. Stratified Analyses for the Association Between Respiratory Syncytial Virus Vaccination and the Risks of Adverse Neonatal Outcomes by Gestational Age at Birth

	EHR evidence of maternal RSV vaccination Event (%)	No EHR evidence of maternal RSV vaccination Event (%)	p-value
**NICU admission**			
<35 weeks' gestation	12 (100.0)	47 (100.0)	NA
≥35 weeks' gestation	77 (7.7)	112 (5.9)	0.05
**Respiratory distress with NICU admission**			
<35 weeks' gestation	7 (58.3)	30 (63.8)	0.70
≥35 weeks' gestation	49 (4.9)	65 (3.4)	0.05
**Jaundice/Hyperbilirubinemia**			
<35 weeks' gestation	10 (83.3)	35 (74.5)	0.70
≥35 weeks' gestation	181 (18.1)	327 (17.1)	0.50
**Hypoglycemia**			
<35 weeks' gestation	3 (25.0)	16 (34.0)	0.70
≥35 weeks' gestation	56 (5.6)	117 (6.1)	0.60
**Sepsis**			
<35 weeks' gestation	0 (0.0)	3 (6.4)	>0.90
≥35 weeks' gestation	3 (0.3)	6 (0.3)	>0.90

RSV=respiratory syncytial virus, NICU=neonatal intensive care unit

© 2024 Son M et al. *JAMA Network Open.*
