# Supplementary material for: Nonadjuvanted Bivalent Respiratory Syncytial Virus Vaccination and Perinatal Outcomes
Source: JAMA Netw Open. 2024 Jul 8;7(7):e2419268. doi: 10.1001/jamanetworkopen.2024.19268 (PMC11231799; doi:10.1001/jamanetworkopen.2024.19268)
Supplement: Supplement 2. — Data Sharing Statement [file jamanetwopen-e2419268-s002.pdf]

# Data Sharing Statement

Son. Nonadjuvanted Bivalent Respiratory Syncytial Virus Vaccination and Perinatal Outcomes. *JAMA Netw Open*. Published July 08, 2024. doi:10.1001/jamanetworkopen.2024.19268

**Data**
**Data available:** Yes
**Data types:** Deidentified participant data, Data dictionary
**How to access data:** mos7003@med.cornell.edu
**When available:** With publication

**Supporting Documents**
**Document types:** None

**Additional Information**
**Who can access the data:** Researchers whose proposed use of the data has been approved.
**Types of analyses:** Research
**Mechanisms of data availability:** With investigator support after approval of a proposal and with a signed data access agreement
